# Black Esophagus: A Rare Case of Acute Esophageal Necrosis

**DOI:** 10.7759/cureus.52660

**Published:** 2024-01-21

**Authors:** Siddharth Chinta, Abhilasha Jyala, Haider Ghazanfar, Jasbir Makker

**Affiliations:** 1 Internal Medicine, BronxCare Hospital Center, Bronx, USA; 2 Gastroenterology, BronxCare Hospital Center, Bronx, USA

**Keywords:** necrotizing esophagitis, hematemesis, esophagus, black esophagus, acute esophageal necrosis (aen)

## Abstract

Acute esophageal necrosis (AEN) also known as necrotizing esophagitis or black esophagus is an extremely rare cause of upper gastrointestinal (GI) bleeding. This condition is considerably rare, and the exact pathophysiology of the development of AEN is still unclear. There is consensus that it is caused by a combination of esophageal mucosal injury due to gastric acid and ischemic injury due to vascular compromise. The management of AEN includes correcting the multitude of underlying predisposing conditions as well as agile symptomatic management and close monitoring for signs of hemodynamic compromise. We here present an interesting case of a middle-aged male patient who presented with hematemesis and underwent emergent esophagogastroduodenoscopy (EGD), which revealed severe necrotic esophagus. We also discussed the risk factors, pathophysiology, and management of AEN.

## Introduction

Acute esophageal necrosis (AEN), also known as necrotizing esophagitis or black esophagus, is an extremely rare cause of upper gastrointestinal (GI) bleeding. It is commonly referred to as black esophagus due to the conspicuous appearance of the esophageal mucosa, which has a friable, patchy, circumferential black presentation up to the gastroesophageal (GE) junction, where it abruptly transitions. This condition is considerably rare with a prevalence estimated to be ranging between 0.01% and 0.2% based on retrospective autopsy studies done 30 years apart [1,2]. Although its exact etiology is unknown, it has been observed that AEN usually is predisposed by a combination of gastric outlet obstruction and vascular compromise [3]. AEN Is associated with an estimated mortality exceeding 30% and requires prompt management for good outcomes [4,5].

## Case presentation

We here present a case of a 56-year-old male patient who presented to the emergency department from the nursing home due to multiple episodes of large-volume hematemesis since morning. His past medical history was significant for untreated hepatitis C, human immunodeficiency virus (HIV), and vascular dementia. His social history was significant for cigarette smoking and alcohol and cocaine use.

At the time of arrival at the emergency department, his heart rate was 104 beats per minute, blood pressure was 110/60 mmHg, temperature was 37.8 °C, and respiratory rate was 18 breaths per minute. He was diaphoretic and lethargic on general examination. He had bilateral temporal wasting and bilateral vesicular breath sounds on lung auscultation. Cardiovascular examination was significant for tachycardia with normal heart sounds. Abdominal examination revealed no distention, normal bowel sounds, no abdominal tenderness, and no hepatosplenomegaly. The patient was intubated and placed on ventilator support. His initial laboratory findings have been mentioned in Table 1.

**Table 1:** Initial laboratory findings MCV: mean corpuscular volume

Laboratory Parameter	Value	Reference Range
White Blood Cell Count	6.4	4.8 - 10.8 k/ul
Hemoglobin	10.7	12.0 - 16.0 g/dl
Hematocrit	31.7	42.0 - 51.0%
MCV	87.5	80.0 - 96.0 fL
Platelet	235	150 - 400 k/ul
Sodium	135	135 - 145 mEq/L
Potassium	5.2	3.5 - 5.0 mEq/L
Bicarbonate	22.0	24 - 30 mEq/L
Chloride	103	98 - 108 mEq/L
Glucose	122	70 - 120 mg/dL
Blood Urea Nitrogen	23.0	8.0 - 26.0 mg/dL
Creatinine	1.9	0.5 - 1.5 mg/dL
Albumin	2.2	3.4 - 4.8 g/dl
Total Bilirubin	0.4	0.2 - 1.2 mg/dL
Direct Bilirubin	0.3	0.0 - 0.3 mg/dL
Alkaline Phosphatase	56	53 - 128 unit/L
Aspartate Transaminase	67	9 - 48 unit/L
Alanine Aminotransferase	19	5 - 40 unit/L
Total Protein	6.3	6.0 - 8.5 g/dl

**Figure 1:**
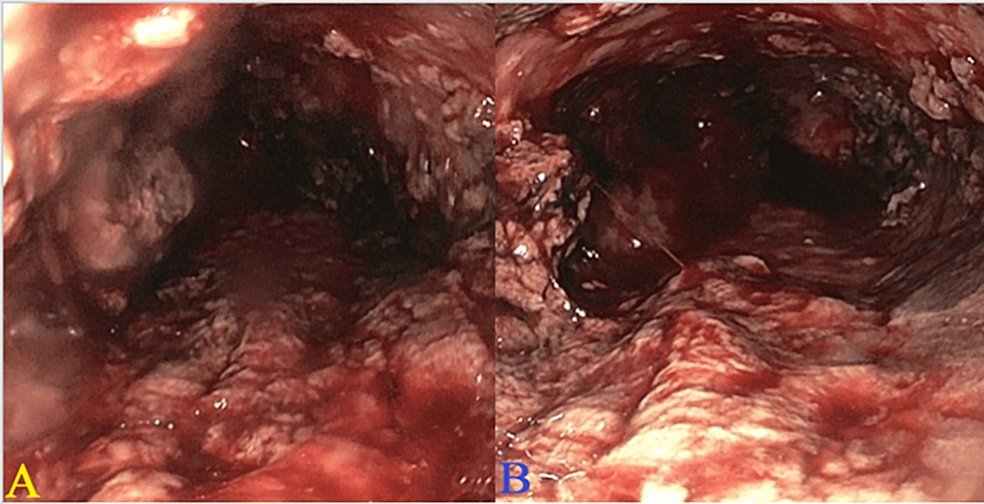
EGD done on day 1 of admission showing necrotic esophagitis with stigmata of recent bleeding in A: middle third of the esophagus and B: lower third of the esophagus EGD: esophagogastroduodenoscopy

He was started on intravenous pantoprazole and fluids. Intravenous octreotide was also administered due to an unclear history of cirrhosis. His chest X-ray was unremarkable. Ultrasound abdomen showed fatty liver and cholelithiasis. He underwent an emergent esophagogastroduodenoscopy (EGD), which revealed severe necrosis in the entire esophagus with stigmata of recent bleeding and red blood in the stomach. His gastric mucosa appeared normal, and no active bleeding was identified in the stomach. There were no gross lesions seen in the duodenal bulb and the second portion of the duodenum. This is shown in Figure 1.

**Figure 2:**
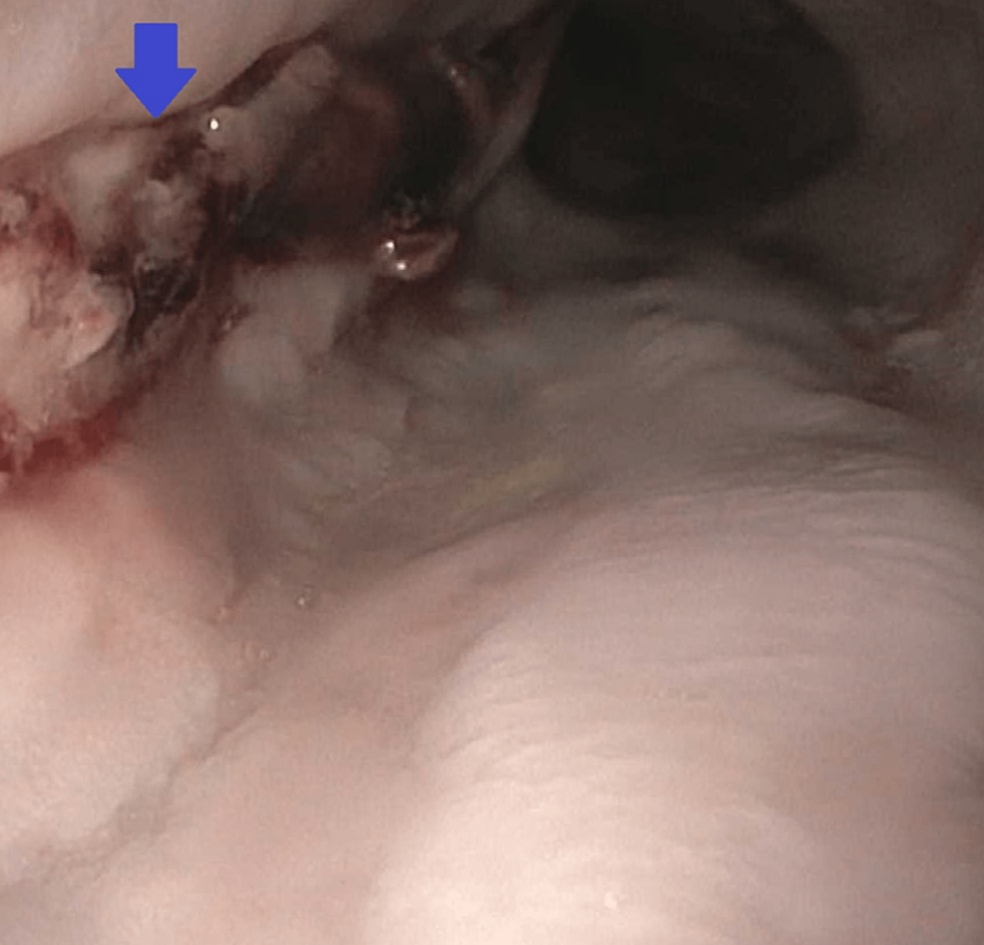
EGD done on day 7 of admission showing one linear esophageal ulcer with stigmata of recent bleeding in the lower one-third of the esophagus EGD: esophagogastroduodenoscopy

He was given a packed red blood cell transfusion for anemia and was continued on an intravenous pantoprazole drip. His clinical condition improved, and he was extubated on day 4 of admission. The patient underwent repeat EGD on day 7 of admission to assess the healing. Repeat EGD showed one linear esophageal ulcer with stigmata of recent bleeding in the lower third of the esophagus, a biopsy of which showed inflammatory changes. This is shown in Figure 2. He was also found to have one nonbleeding gastric ulcer with a clean base (Forrest Class 3).

His hemoglobin remained stable, and he had no repeat episodes of hematemesis. The patient was continued on an oral proton pump inhibitor and was discharged in stable condition with a plan to repeat EGD to check for healing after two to three months as an outpatient.

## Discussion

AEN is predominantly seen in elderly male patients with multiple comorbidities and poor nutritional status [6]. Chronic alcohol use, illicit drug use, malignancy, solid organ transplantation, various infectious states causing immunocompromise, including human immunodeficiency virus (HIV), herpes simplex virus (HSV), cytomegalovirus (CMV), and fungal infections have been associated with diffuse AEN [7,8]. Our patient had multiple risk factors for AEN such as untreated hepatitis C, HIV, and a history of cigarette smoking, alcohol use, and cocaine use [7].

Although the exact pathophysiology of the development of AEN is still unclear, there is consensus that it is caused by a combination of esophageal mucosal injury due to gastric acid and ischemic injury due to vascular compromise [9]. The vascular supply of the esophagus is unique in that the distal esophagus is relatively sparingly vascularized compared to the proximal and middle portions. Therefore, distal esophageal involvement is seen classically with AEN. Although the appearance of the esophageal mucosa in AEN is characterized by patchy, friable, blackish discoloration that is circumferential and typically involves the distal esophagus, a biopsy of the mucosa is necessary to differentiate AEN from other conditions that appear with darkish discoloration of the esophageal mucosa and to establish etiology wherein AEN is predisposed by an acute infectious process [10-12].

Uncomplicated AEN follows a predictable disease course that is characterized by an initial phase with a healthy viable esophageal mucosa, followed by the acute disease state which is usually when the patient has clinical symptoms such as hematemesis and melena with endoscopic findings of circumferential necrotic discoloration starting at the GE junction and extending proximally [13]. Although it is typically confined to the distal esophagus in most cases, proximal esophageal involvement has also been observed. The histopathological analysis is significant for marked necrotic changes, substantial leukocytic infiltrate, and a notable absence of squamous epithelium. The last stage of the disease occurs with the onset of healing by the regeneration of the mucosal epithelial lining. There are whitish exudates that are seen in between scattered areas of blackish discoloration, which gives a chessboard-like appearance on endoscopic inspection. AEN is commonly associated with complications such as esophageal perforation, strictures, and life-threatening bleeding, which collectively contribute to the high rate of mortality and morbidity [14].

The management of AEN includes correcting the multitude of underlying predisposing conditions as well as agile symptomatic management and close monitoring for signs of hemodynamic compromise. The role of intravenous crystalloids is paramount in volume expansion and remedying the low flow state, which is an inciting factor in the disease process. Blood products should be transfused to maintain appropriate blood counts [15]. Proton pump inhibitors (PPI) should be used promptly to cause gastric acid suppression, which can reduce the injury to the mucosal lining [6]. Sucralfate can be considered for increased efficacy in the absence of relative contraindications [16]. Though oral intake is held off for the first 24 hours, there is ambiguity on the timing of resuming enteral nutrition [17]. Agitation of the friable necrotic mucosa with the use of naso/orogastric tubes should be avoided to minimize complications such as further bleeding and perforation [18]. Bleeding should be rapidly controlled using submucosal epinephrine and expanding stents. Balloon tamponade should be avoided due to the risk of perforation. The role of prophylactic antibiotics is unclear and should be used in the setting of immunocompromised states and positive cultures although anecdotal reports suggest that indiscreet usage of broad-spectrum antimicrobials has been associated with the progression of AEN [19]. Perforations and strictures should be managed either surgically or with an expanding stent if surgery is not feasible [18].

The outcomes are generally poor with AEN, but in the absence of critical illness and multiple comorbidities, there are reasonable chances for recovery in healthy individuals. Repeat endoscopy should be performed to assess for healing and to guide further treatment. Again, the consensus for the timing of the endoscopy is unclear and can vary from as early as the first week, like our case, to up to a few months [3,20].

## Conclusions

AEN is a lethal syndrome that presents as upper gastrointestinal bleeding and is diagnosed by endoscopy. It is associated with a significantly high mortality rate. Due to its rare presentation, there is no clear consensus on managing AEN, and anecdotal data show that symptomatic treatment, including intravenous fluid resuscitation and management of underlying commodities, is associated with favorable outcomes and decreased complications.
